# Is radiotherapy still the optimal initial choice for patients with early-stage low-grade follicular lymphoma in the modern era? A population-based study

**DOI:** 10.1007/s00277-024-06022-1

**Published:** 2024-09-28

**Authors:** Wenshuai Zheng, Shenyu Wang, Yanchao Liang, Hongmei Ning

**Affiliations:** 1 Department of Hematology, Hainan Hospital of Chinese PLA General Hospital, Sanya, 572000 Hainan China; 2 Senior Department of Hematology, Fifth Medical Center of Chinese PLA General Hospital, Beijing, 100071 China

**Keywords:** Follicular lymphoma, Radiotherapy, Survival, Second primary malignancies, SEER, Population-based

## Abstract

Despite radiotherapy (RT) is recognized as preferred initial therapy for early-stage low-grade follicular lymphoma (FL) by many international practice guidelines, the medical oncologist has improperly underutilized RT, and diverse management strategies, including systemic therapy (ST), combined modality (CM) and watch and wait (WW), are still used. Except survival outcomes, previous studies concerned little about the treatment-related toxicity, which is also important factor in choosing initial management strategy, especially second primary malignancies (SPMs). The aim of this study was to compare the overall survival (OS) and the SPMs risk between different management strategies, which can provide guidance for the choice of optimal initial management strategy. Data was acquired from the Surveillance, Epidemiology, and End Results (SEER) database. Finally, A total 10,900 patients were identified, in which 930 cases developed SPMs. The use of radiotherapy (RT) has remained consistently low, with a utilization rate of around 20%, while most patients have received watchful waiting (WW) and systemic therapy (ST). In the rituximab era, multivariate analysis indicated that RT exhibited significantly superior OS and did not increase SPMs risk in comparison with ST and WW. At the same time, although there were no significant differences in OS between CM and RT, RT had significantly lower SPMs risk in comparison with CM. The use of RT improved the OS and did not increase the SPMs risk in comparison with other management strategies. Considering the low application rate of RT, oncologists should emphasize and increase the use of RT as an initial management strategy in patients with early-stage low-grade FL.

**Supplementary Information:**

The online version contains supplementary material available at 10.1007/s00277-024-06022-1.

## Introduction

Follicular lymphoma (FL) is the most common subtype of indolent non-Hodgkin lymphoma (NHL). It is characterized by the hallmark translocation t(14;18) which results in the overexpression of the BCL-2 protein, a member of a family of proteins that blocks apoptosis [1]. Although the majority of patients with FL present advanced-stage (stage III-IV) disease, approximately 25–30% have early-stage (stage I-II) disease at diagnosis [2]. Since FL is highly radiosensitive, radiotherapy (RT) alone to the early-stage FL can achieve long-term disease control with the 10-year PFS and OS rates ranging from 40 to 59% and 58–86%, respectively [3–6]. As such, National Comprehensive Cancer Network (NCCN) and European Society of Medical Oncology (ESMO) guidelines have recommend RT as preferred management strategy for early-stage low-grade FL for many years [7, 8]. However, despite the existence of guidelines that recommend RT, the RT use for patients with early-stage low-grade FL is underutilized [3, 9, 10]. Conversely, more patients received other management strategies, including systemic therapy (ST) (chemotherapy, immunotherapy or chemoimmunotherapy), combined modality (CM) and watch and wait (WW), as initial therapy [10, 11]. The reason for this phenomenon is the lack of the high-quality evidence of optimal initial management strategy for early-stage low-grade FL. Although many studies had explored the survival disparities between different initial management strategies in early-stage low-grade FL, the results of those studies are inconsistent and there are rare randomized controlled trials [3, 11–14]. In addition, treatment-related toxicity is also important factor in choosing initial management strategy, especially second primary malignancies (SPMs). This could be unacceptable in early-stage FL characterized by good prognosis and few symptoms. Because SPMs not only be the third most common cause of death in FL patients treated in the rituximab era [15], but also increase the economic burden of treatment. However, there are few studies comparing the SPMs risk between different initial management strategies.

To provide data regarding the comparison of the OS and the SPMs risk between different initial management strategies in early-stage low-grade FL, we conducted this large, population-based analysis based on Surveillance, Epidemiology, and End Results (SEER) database. This will provide further evidences for the choice of optimal initial management strategy in early-stage low-grade FL.

## Patients and methods

### Patients

The data of this study were obtained from the National Cancer Institute’s SEER database, which was an authoritative source of population-based cancer statistics in the United States (U.S.) and currently covers about ~ 30% of the U.S. population [16]. Our analysis was limited to SEER 17 registries database, released April 2023 based on the November 2022 submission.

We identified and categorized patients with FL using the third edition of the International Classification of Diseases for Oncology (9691 and 9695). The inclusion criteria were (1) age ≥ 18 years, (2) World Health Organization grade 1 to 2 FL, (3) early-stage (stage I-II) FL. We use summary stage for lymphoma to determine the exact stage. The exclusion criteria were (1) FL diagnosis confirmed only by autopsy or death certificate, (2) patients with incomplete survival data, (3) Patients with non-primary malignancies, (4) patients with unknown race/ethnicity (82) and patients with non-Hispanic Native American and Alaskan Natives (47). Because the number of these patients was too small for meaningful statistical analysis, (5) When analyzing SPMs, diffuse large B-cell lymphoma and Burkitt lymphoma were excluded. Because these lymphomas might have been misclassified as SPMs when they may be histological transformation of FL actually [17]. In addition, SPMs detected within the first 6 months after the diagnosis of FL were also excluded, which can avoid ascertainment bias. Patients with known race/ethnicity were grouped into non-Hispanic white (NHW), non-Hispanic black (NHB), non-Hispanic Asian or Pacific Islander (NHA/PI), and Hispanic (regardless of races). Age was divided into three age groups: 18–59, 60–69 and ≥ 70 years. RT and ST were classified as yes and none/unknown. The SEER database records first course of therapy data, which is defined as a treatment plan initiated within 12 months of diagnosis. Second-line therapies are not recorded. Therefore, patients receiving RT and ST simultaneously were defined as patients receiving CM as initial management strategy and patients receiving neither RT nor ST were defined as patients receiving WW as initial management strategy. The year of diagnosis of FL was used as surrogate for the evolution of treatment paradigm of RT and ST due to the absence of actual treatment data in the SEER database. We divided patients into two eras: 2000–2008 (herein referred to as Era1) represented pre-rituximab and high-dose extensive-field RT era, and 2009–2020 (herein referred to as Era2) represented rituximab and low-dose limited-field RT era for early-stage low-grade FL.

### Statistical analysis

The OS was calculated from the time of FL diagnosis to the time of death or last follow-up, whichever comes first. The OS was calculated with Kaplan-Meier analysis and the statistical significance of OS between different variables was determined by the log-rank test. To evaluate the impact of different initial management strategies on OS, we used multivariate Cox proportional hazards model, which was adjusted through stratification for era, gender, stage, age, grade, extranodal disease, race/ethnicity, to calculate hazard ratios (HR) and 95% confidence interval (CI).

SPMs was defined as the first subsequent primary malignancy occurring at least 6 months after FL diagnosis. This definition can reduce the potential for detection bias, as the likelihood of incidental cancer detection is highest immediately following FL diagnosis. Follow-up for analysis of SPMs started from 6 months after FL diagnosis to SPMs development, death or last follow-up, whichever comes first. We estimated relative risk (RR) and 95% CIs of SPMs according to the receipt of initial management strategy using multivariate Poisson regression model that was adjusted through stratification for era, gender, stage, age, grade, extranodal disease, race/ethnicity and follow up time.

Data were extracted using SEER*Stat (version 8.4.1.2). Statistical analysis was conducted using R 4.2.3 software (R Development Core Team). All *P* values were calculated as two-sided and *P* values less than 0.05 were considered statistically significant.

## Results

### Patients’ characteristics

Finally, 10,900 patients with early-stage low-grade FL were identified during 2000–2020. Of those, most patients were stage I (*n* = 6802, 62.4%), aged 18–59 years (*n* = 4296, 39.4%), grade 2 (*n* = 6118, 56.1%), no extranodal disease (*n* = 8629, 79.2%), and NHW (*n* = 8670, 79.5%). Overall, 2294 (21.0%) patients received RT only, 2832 (26.0%) patients received ST only, 564 (5.2%) patients received CM, and 5210 (47.8%) patients received WW. The baseline for all patients are summarized in Table 1.

**Table 1:** Patients demographics and clinical characteristics

Clinical features	Overall (*n* = 10900)	RT (*n* = 2294)	ST (*n* = 2832)	CM (*n* = 564)	WW (*n* = 5210)
Era, n (%)					
Era1	4554 (41.8)	937 (40.8)	1293 (45.7)	343 (60.8)	1981 (38.0)
Era2	6346 (58.2)	1357 (59.2)	1539 (54.3)	221 (39.2)	3229 (62.0)
Age, n (%)					
18–59	4296 (39.4)	984 (42.9)	1246 (44.0)	293 (52.0)	1773 (34.0)
60–69	2966 (27.2)	650 (28.3)	774 (27.3)	146 (25.9)	1396 (26.8)
≥ 70	3638 (33.4)	660 (28.8)	812 (28.7)	125 (22.2)	2041 (39.2)
Gender, n (%)					
Female	5480 (50.3)	1126 (49.1)	1439 (50.8)	252 (44.7)	2663 (51.1)
Male	5420 (49.7)	1168 (50.9)	1393 (49.2)	312 (55.3)	2547 (48.9)
Stage, n (%)					
Stage I	6802 (62.4)	1896 (82.7)	1155 (40.8)	366 (64.9)	3385 (65.0)
Stage II	4098 (37.6)	398 (17.3)	1677 (59.2)	198 (35.1)	1825 (35.0)
Grade, n (%)					
Grade 1	4782 (43.9)	983 (42.9)	1222 (43.1)	203 (36.0)	2374 (45.6)
Grade 2	6118 (56.1)	1311 (57.1)	1610 (56.9)	361 (64.0)	2836 (54.4)
Extranodal disease, n (%)					
No	8629 (79.2)	1717 (74.8)	2383 (84.1)	448 (79.4)	4081 (78.3)
Yes	2271 (20.8)	577 (25.2)	449 (15.9)	116 (20.6)	1129 (21.7)
Race/ethnicity, n (%)					
NHB	408 (3.7)	56 (2.4)	113 (4.0)	18 (3.2)	221 (4.2)
NHW	8670 (79.5)	1851 (80.7)	2230 (78.7)	449 (79.6)	4140 (79.5)
NHA/PI	568 (5.2)	161 (7.0)	131 (4.6)	24 (4.3)	252 (4.8)
Hispanic	1254 (11.5)	226 (9.9)	358 (12.6)	73 (12.9)	597 (11.5)

NHW: non-Hispanic white; NHB: non-Hispanic black; NHA/PI: non-Hispanic Asian or Pacific Islander; RT: radiotherapy; ST: systemic therapy; CM: combined modality; WW: watch and wait

### Trends in the application of different management strategies

**Fig. 1:**
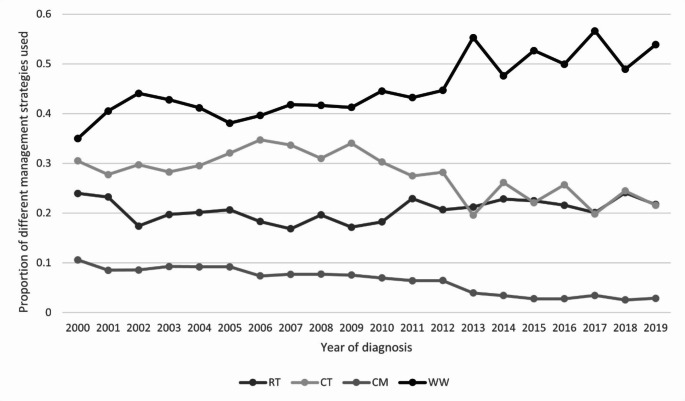
Trends in the application of different management strategies. RT: radiotherapy; ST: systemic therapy; CM: combined modality; WW: watch and wait

Trends in the percentage utilization for various management strategies as a function of time are summarized in Fig. 1. Overall, RT use has remained stable at around 20% during 2000–2019. Before 2009, ST and WW use has remained stable at around 30% and 45% respectively. After 2009, there was a notable decline in ST use, from 34% in 2009 to only 22% in 2019. Correspondingly, there was a 12% absolute increase in the WW use, from 43% in 2009 to 55% in 2019. CM use declined gradually from 10 to 3% during 2000–2019.

### Survival analysis

**Fig. 2:**
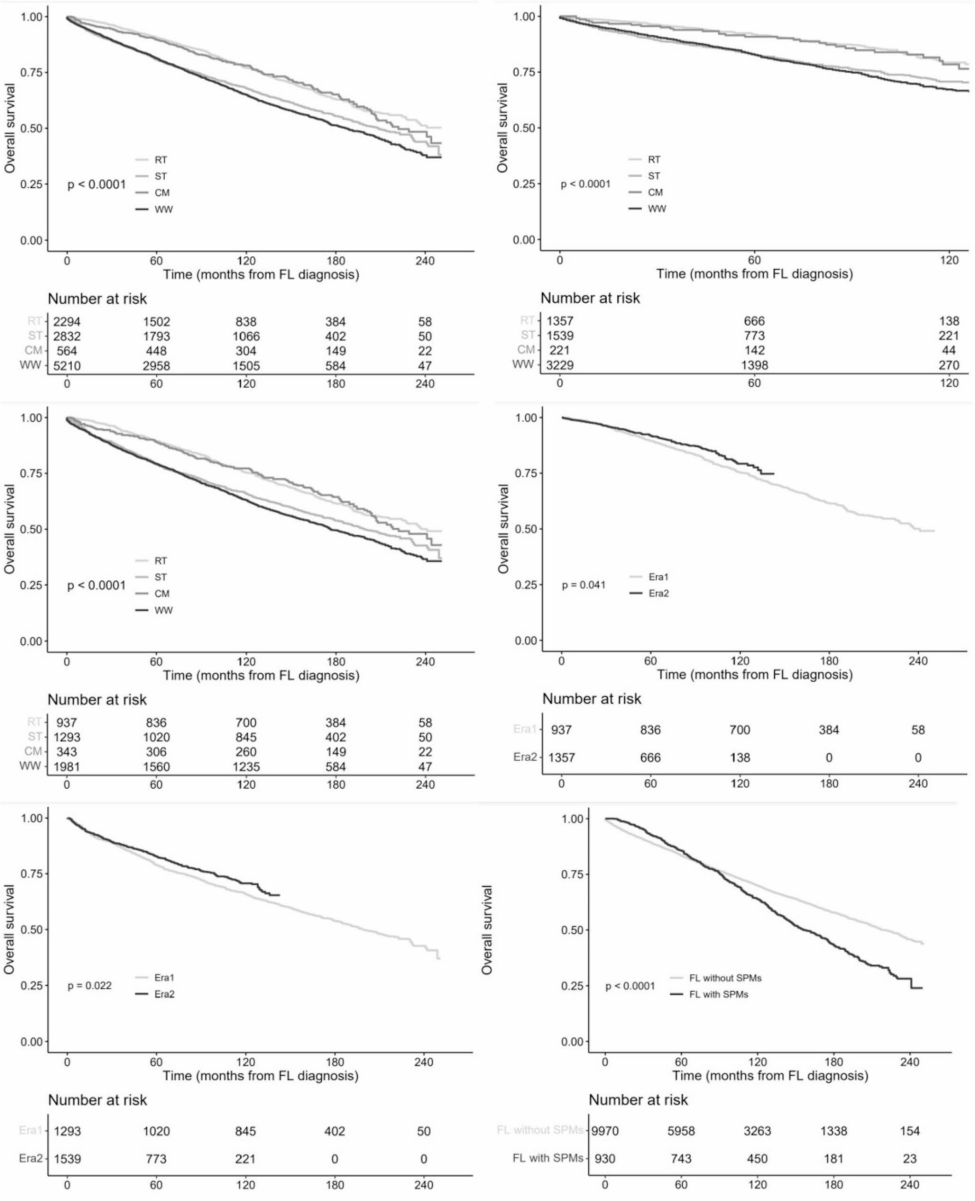
Kaplan–Meier curves by management strategy in (**A**) All patients, (**B**) Patients diagnosed in Era1, (**C**) Patients diagnosed in Era2. Kaplan–Meier curves by era of diagnosed in (**D**) Patients receiving radiotherapy, (**E**) Patients receiving systemic therapy. Kaplan–Meier curves in (**F**) Patients with or without SPMs. FL: follicular lymphoma; RT: radiotherapy; ST: systemic therapy; CM: combined modality; WW: watch and wait; SPMs: second primary malignancies

There were a total 3272 deaths in the entire cohort, with an estimated median OS of 207 months (range, 0-251 months) and a 5-year, 10-year and 15-year OS rates of 83.5% (95% CI, 82.7-84.2%), 69.1% (95% CI, 68.1-70.2%), and 55.8% (95% CI, 54.5-57.1%), respectively. The results of multivariate Cox proportional hazards analysis of OS were summarized in Table 2 and the survival curves were showed in Fig. 2. For all patients, patients receiving ST (Log-rank: *P* < 0.001; multivariate: HR, 1.414; *P* < 0.001) or WW (Log-rank: *P* < 0.001; multivariate: HR, 1.315; *P* < 0.001) exhibited significantly poorer OS in comparison with patients receiving RT. Patients receiving CM (Log-rank: *P* < 0.001; multivariate: HR, 1.168; *P* = 0.076) exhibited similar OS in comparison with patients receiving RT. For patients receiving RT or ST, patients diagnosed in Era2 (RT: Log-rank, *P* = 0.041; multivariate, *P* = 0.014, ST: Log-rank, *P* = 0.022; multivariate, *P* = 0.014) exhibited significantly superior OS in comparison with patients diagnosed in Era1 (Fig. 2(D), 2(E); Table S1). Considering the changes in OS in the two eras of patients receiving RT or ST, we conducted a subgroup analysis according to era. No matter for patients diagnosed in Era1 or Era2, the results of survival comparison between different management strategies were similar to the results of all patients’ survival comparison between different management strategies (Table 2).

**Table 2:** Multivariate analysis of overall survival

Variable	All patients		Patients diagnosed in Era1		Patients diagnosed in Era2	
	Hazard ratio (95% CI)	*P*	Hazard ratio (95% CI)	*P*	Hazard ratio (95% CI)	*P*
Era						
Era1	Reference					
Era2	0.845 (0.779–0.916)	< 0.001				
Age, years						
18–59	Reference		Reference		Reference	
60–69	2.435 (2.180–2.719)	< 0.001	2.540 (2.231–2.890)	< 0.001	2.231 (1.803–2.759)	< 0.001
≥ 70	7.880 (7.152–8.682)	< 0.001	7.641 (6.812–8.572)	< 0.001	8.483 (7.058–10.196)	< 0.001
Gender						
Female	Reference		Reference		Reference	
Male	1.245 (1.161–1.333)	< 0.001	1.265 (1.162–1.376)	< 0.001	1.199 (1.063–1.353)	0.003
Stage						
Stage I	Reference		Reference		Reference	
Stage II	1.147 (1.065–1.235)	< 0.001	1.100 (1.004–1.205)	0.041	1.233 (1.086-1.400)	0.001
Grade						
Grade 1	Reference		Reference		Reference	
Grade 2	0.994 (0.927–1.065)	0.858	0.948 (0.871–1.032)	0.216	1.101 (0.972–1.246)	0.130
Extranodal disease						
No	Reference		Reference		Reference	
Yes	0.854 (0.783–0.931)	< 0.001	0.883 (0.796–0.979)	0.018	0.806 (0.688–0.943)	0.007
Race/ethnicity						
NHB	Reference		Reference		Reference	
NHW	0.794 (0.662–0.951)	0.012	0.788 (0.630–0.985)	0.036	0.774 (0.567–1.057)	0.107
NHA/PI	0.634 (0.493–0.814)	< 0.001	0.519 (0.375–0.720)	< 0.001	0.824 (0.552–1.229)	0.343
Hispanic	0.775 (0.623–0.964)	0.022	0.826 (0.627–1.089)	0.174	0.699 (0.487–1.002)	0.051
Therapy						
RT	Reference		Reference		Reference	
ST	1.414 (1.265–1.581)	< 0.001	1.280 (1.121–1.461)	< 0.001	1.797 (1.459–2.214)	< 0.001
CM	1.168 (0.984–1.387)	0.076	1.110 (0.916–1.346)	0.287	1.219 (0.828–1.793)	0.316
WW	1.315 (1.190–1.453)	< 0.001	1.178 (1.046–1.326)	< 0.007	1.690 (1.402–2.038)	< 0.001

CI: confidence interval; NHW: non-Hispanic white; NHB: non-Hispanic black; NHA/PI: non-Hispanic Asian or Pacific Islander; RT: radiotherapy; ST: systemic therapy; CM: combined modality; WW: watch and wait

### SPMs analysis

Among the 10,900 identified patients, 10,101 patients were ≥ 6-month survivors (Table S2), and 930 cases developed SPMs at various sites. SPMs were diagnosed at a median time of 48 months (range, 6-244 months) post-FL diagnosis. The cumulative incidence of SPMs was 6.6% (95% CI, 6.0-7.1%), 11.2% (95% CI, 10.5-12.0%) and 15.7% (95% CI, 14.5-16.8%) at 5, 10 and 15 years, respectively. The results of multivariate Poisson regression analysis of SPMs risk were summarized in Table 3. For all patients, patients receiving ST (multivariate: RR, 0.900; *P* = 0.288), WW (multivariate: RR, 0.869; *P* = 0.107) or CM (multivariate: RR, 1.123; *P* = 0.417) had similar SPMs risk in comparison with patients receiving RT. For patients receiving RT or ST, multivariate analysis showed that patients diagnosed in Era2 (RT: multivariate, RR, 0.174; *P* < 0.001, ST: multivariate, RR, 0.365; *P* < 0.001) were associated with a decreased SPMs risk in comparison with patients diagnosed in Era1 (Tabel S3). So, we also conducted a subgroup analysis according to era. For patients diagnosed in Era1, patients receiving ST (multivariate, RR, 0.719; *P* = 0.006) had significantly lower SPMs risk in comparison with patients receiving RT. Patients receiving WW (multivariate, RR, 0.813; *P* = 0.053) or CM (multivariate, RR, 0.867; *P* = 0.396) had similar SPMs risk in comparison with patients receiving RT. For patients diagnosed in Era2, patients receiving CM (multivariate, HRRR, 2.006; *P* = 0.010) had significantly higher SPMs risk in comparison with patients receiving RT. Patients receiving WW (multivariate, RR, 1.038; *P* = 0.806) or ST (multivariate, RR, 1.364; *P* = 0.073) had similar SPMs risk in comparison with patients receiving RT. For the survival, patients with SPMs had significant inferior OS in comparison with patients without SPMs (log-rank, *P* < 0.001, Fig. 2(F)). The separation of survival curves occurred approximately 8 years after the diagnosis of FL, which was near 4 year later than the median time of SPMs diagnosis.

**Table 3:** Multivariate analysis of second primary malignancies risk

Variable	All patients		Patients diagnosed in Era1		Patients diagnosed in Era2	
	Relative risk (95% CI)	*P*	Relative risk (95% CI)	*P*	Relative risk (95% CI)	*P*
Era						
Era1	Reference					
Era2	0.301 (0.261–0.348)	< 0.001				
Age, years						
18–59	Reference		Reference		Reference	
60–69	1.190 (1.016–1.394)	0.031	1.185 (0.977–1.436)	0.084	1.192 (0.902–1.573)	0.216
≥ 70	0.862 (0.733–1.014)	0.073	0.634 (0.514–0.780)	< 0.001	1.346 (1.033–1.757)	0.028
Gender						
Female	Reference		Reference		Reference	
Male	1.270 (1.115–1.447)	< 0.001	1.166 (0.991–1.374)	0.064	1.404 (1.128–1.751)	0.002
Stage						
Stage I	Reference		Reference		Reference	
Stage II	0.971 (0.843–1.117)	0.682	0.978 (0.820–1.163)	0.801	0.948 (0.747-1.200)	0.659
Grade						
Grade 1	Reference		Reference		Reference	
Grade 2	0.975 (0.854–1.113)	0.707	1.041 (0.885–1.224)	0.624	0.904 (0.723–1.135)	0.380
Extranodal disease						
No	Reference		Reference		Reference	
Yes	1.105 (0.941–1.293)	0.217	1.133 (0.926-1/376)	0.217	1.076 (0.818–1.397)	0.592
Follow up time, months						
6–59	Reference		Reference		Reference	
≥ 60	0.305 (0.265–0.351)	< 0.001	0.235 (0.199–0.278)	< 0.001	0.488 (0.382–0.618)	< 0.001
Race/ethnicity						
NHB	Reference		Reference		Reference	
NHW	0.970 (0.707–1.374)	0.855	0.892 (0.605–1.383)	0.585	1.114 (0.665–2.049)	0.705
NHA/PI	0.766 (0.484–1.212)	0.253	0.837 (0.472–1.486)	0.542	0.676 (0.310–1.475)	0.319
Hispanic	0.650 (0.438–0.981)	0.036	0.680 (0.410–1.149)	0.140	0.648 (0.344–1.290)	0.194
Therapy						
RT	Reference		Reference		Reference	
ST	0.900 (0.742–1.093)	0.288	0.719 (0.568–0.911)	0.006	1.364 (0.974–1.920)	0.073
CM	1.123 (0.843–1.478)	0.417	0.867 (0.618–1.196)	0.396	2.006 (1.149–3.330)	0.010
WW	0.869 (0.734–1.032)	0.107	0.813 (0.661–1.005)	0.053	1.038 (0.776–1.404)	0.806

CI: confidence interval; CI: confidence interval; NHW: non-Hispanic white; NHB: non-Hispanic black; NHA/PI: non-Hispanic Asian or Pacific Islander; RT: radiotherapy; ST: systemic therapy; CM: combined modality; WW: watch and wait

## Discussion

To our best knowledge, other than survival analysis, this is the largest series of analysis comparing the SPMs risk between different management strategies in early-stage low-grade FL.

Although international clinical practice guidelines endorse RT as the preferred initial management strategy for patients with early-stage low-grade FL [7, 18], the majority of patients did not follow those guidelines. In the SEER database, only 34% of patients with stage I-II FL received RT as initial or combined treatment [3]. In the National LymphoCare Study (NLCS), only 23% of patients with stage I FL received RT [9]. Furthermore, the National Cancer Database showed a 13% decrease in the use of RT, from 37% in 1999 to 24% in 2012 [10]. In our study, the utilization of RT steadily remained at low levels with only a rate around 20%. This result is consistent with those of previous studies. At the same time, most patients received WW or ST. After 2009, there was a notable increase in the number of WW use with a corresponding decrease in the number of ST use. In addition, A small number of patients received CM and it’s use declined gradually from 11 to 3% during 2000–2019. These data suggest that RT use for early-stage low-grade FL has been underused.

Before 2008, CHOP-based induction chemotherapy (cyclophosphamide, doxorubicin, vincristine, prednisone) was the standard treatment paradigm for most patients with early-stage low-grade FL, who received ST. With the arrival of rituximab and its efficacy and tolerability in advanced disease, the status of chemotherapy was replaced by immunotherapy with or without chemotherapy in early-stage low-grade FL [19]. For patients receiving ST, our study showed that patients diagnosed in Era2 had significantly superior survival and lower SPMs risk in comparison with patients diagnosed Era1. This result indicated that the immunotherapy improved the survival and decreased the SPMs risk in comparison with chemotherapy for patients receiving ST. For patients receiving RT, the change of the survival and the SPMs risk between two eras is similar to the pattern of patients receiving ST. This may be attributed to the change of usage model of RT. In Era1, the usage of RT was usually large dose, up to 50 Gy, together with large radiation fields, in which both enlarged nodes and healthy surrounding nodes were irradiated [5, 6]. This approach can obtain an excellent disease control but it also induced significant acute and chronic toxicity, such as SPMs [5, 6, 20]. In Era2, 24 Gy and limited radiation fields, including involved field RT (IFRT) and involved site RT (ISRT), was established as the appropriate RT dose and fields for early-stage low-grade FL [21–26]. In addition, the extensive use of positron emission tomography-computed tomography in Era2, which more adequately define early-stage disease, also improved the survival outcome of early-stage FL [27]. Considering the changes in the survival and the SPMs risk in the two eras of patients receiving RT or ST, we conduct a subgroup analysis according to era in following analysis.

For the choice of initial management strategy, WW represents the gold standard in asymptomatic advanced-stage FL [7, 18]. For early-stage FL, a large population-based study demonstrated a significantly reduced OS for patients receiving WW in comparison with patients receiving RT [10]. On the contrary, two prospective studies did not report a worse progress-free survival (PFS) and OS for patients receiving WW in comparison with patients receiving RT [11, 12]. Our study indicated that patients receiving WW exhibited significantly poorer OS in comparison with patients receiving RT, no matter in all patients or patients diagnosed in Era1 or Era2. In pre-rituximab era, there were rarely studies about the use of ST in early-stage FL. In rituximab era, some studies reported that patients receiving chemoimmunotherapy or rituximab alone get better PFS in comparison with patients receiving RT [11, 13, 14, 28]. But the OS had no differences between patients receiving ST and patients receiving RT in all these studies. In our study, patients receiving ST exhibited significantly inferior OS compared to patients receiving RT, regardless of whether they were diagnosed in Era1 or Era2. Considering that a significant proportion of cases relapse after RT and most of them do outside the radiation field [13, 23, 24, 28], many studies explored the rationality of CM. Two studies demonstrated that the combination of IFRT or ISRT and anti-CD20 antibody has led to similar efficacy compared with large field irradiation but with markedly reduced side effects [29, 30]. In a prospective, randomized, phase III trial, in which half of the patients were randomized to IFRT alone versus IFRT combined with CVP (CHOP without doxorubicin) +/- rituximab (rituximab administered together with CVP regimen after 2006), the PFS benefit of patients receiving CM was observed only after the introduction of rituximab. However, there were no significant differences in OS in the 2 cohorts and toxicity was higher in the CM cohort [31]. Two retrospective studies, which compared the survival disparity of IFRT alone versus IFRT plus rituximab, found better PFS with the CM strategy [13, 14], while the OS also had no differences between different treatment groups. Our study showed that there were no significant differences in OS between patients receiving CM and patents receiving RT, which was consistent with previous studies.

With regard to the comparison of SPMs risk between different management strategies, the studies were few. In a prospective single arm study with CM (RT combined with CHOP/CVP + bleomycin), there were 14 SPMs in 102 patients with 10 years of follow-up [32]. A lager retrospective study reported that the incidence of SPMs had no statistically significant differences between different management strategies, including RT, ST, CM and WW [12]. In a population-base study, multivariate analysis showed that patients receiving RT was associate with a higher SPMs risk in comparison with patients receiving other management strategies [33]. In our study, multivariable Poisson regression analysis of all patients showed that there were no significant differences in SPMs risk between different management strategies. When taking a subgroup analysis according to era, there are some differences. For patients diagnosed in Era1, the SPMs risk of patients receiving RT was significantly higher in comparison with patients receiving ST. This may be caused by the way RT is used in Era1 with extended fields and higher dose. For patients diagnosed in Era2, there were no differences in SPMs risk when patients receiving RT were compared with patients receiving ST. Combined with the result that Era2 was associated with a decreased SPMs risk in comparison with Era1 for patients receiving RT or ST. The change of SPMs risk between patients receiving RT and ST in the two eras maybe due to a greater reduction in SPMs risk in patients receiving RT than that in those receiving ST. The decreased SPMs risk of patients receiving RT in Era2 may be attributed mainly to the change of the way RT is used in Era2 with local fields and lower dose, while the decreased SPMs risk of patients receiving ST in Era2 may be attributed mainly to the reduction use of chemotherapy, which was consistent with recent study that demonstrated the relation between doxorubicin and the risk of SPMs in lymphoma [34]. For RT versus CM, our study showed that there were no significant differences in SPMs risk between patients receiving RT and patients receiving CM in Era1. The reason may be that patients receiving RT had significant higher SPMs risk in comparison with patients receiving ST in Era1. So, RT plays a dominant role in SPMs development in CM. Conversely, in Era2, patients receiving RT had similar SPMs risk in comparison with patients receiving ST. Therefore, patients receiving CM should have higher SPMs risk in comparison with patients receiving RT in Era2, due to the similar role of ST and RT in SPMs development in CM. This speculation is consistent with our results, in which patients receiving CM had higher SPMs risk in comparison with patients receiving RT in Era2. Considering that patients with SPMs had inferior survival in comparison with patients without SPMs, the higher SPMs risk of CM may partly explain the reason why CM did not improve the survival in comparison with RT in era2.

Important limitation of the SEER registries is the lack of detailed therapeutic regimens of first course. But we used the year of diagnosis of FL as surrogate for the evolution of treatment paradigm of RT and ST to reduce the bias. The strength of our study is that SEER registries are population-based, avoiding potential selection biases that could arise from institution-based studies. In addition, the large size of the cohort, long-running, and focus on internal cohort comparisons were also key strengths of our study. Because of the rarity and good prognosis of localized FL, studies in this field are primarily hindered by their lengthy completion periods which result in minimal survival benefits and differences in the incidence of SPMs.

In conclusion, we compared the OS and the SPMs risk between different initial management strategies for patients with early-stage low-grade FL based on SEER database. In rituximab era, patients receiving RT had superior OS and did not increase the SPMs risk in comparison with patents receiving ST or WW. At the same time, although there was no significant difference in OS between patients receiving CM and patients receiving RT, patients receiving RT had significantly lower SPMs risk. Given the fact that RT was associated with a survival advantage over ST and WW, and a lower SPMs risk over CM, the use of RT should remain standard-of-care for low-grade early-stage FL. This study provided important evidences to define the optimal initial management strategy in patients with low-grade early-stage FL.

## Electronic supplementary material

Below is the link to the electronic supplementary material.

Supplementary Material 1

## Data Availability

The data were obtained from the SEER database (https://seer.cancer.gov/seerstat/), which is freely accessible to the public.
