# Supplementary material for: Is radiotherapy still the optimal initial choice for patients with early-stage low-grade follicular lymphoma in the modern era? A population-based study
Source: Ann Hematol. 2024 Sep 28;103(11):4589–98. doi: 10.1007/s00277-024-06022-1 (PMC11534986; doi:10.1007/s00277-024-06022-1)
Supplement: Supplementary file 1 — Supplementary Material 1 [file 277_2024_6022_MOESM1_ESM.docx]

**Is radiotherapy still the optimal initial choice for patients with** **early-stage low-grade follicular lymphoma in the modern era? a** **population-based study**

Wenshuai Zheng^1^^＃^, Shenyu Wang^2#^, Yanchao Liang^1＃^, Hongmei Ning^2^

^1^Department of Hematology, Hainan Hospital of Chinese PLA General Hospital, Sanya, Hainan 572000, China

^2^Senior Department of Hematology, Fifth Medical Center of Chinese PLA General Hospital, Beijing 100071, China

Corresponding Author: Dr. Hongmei Ning, Senior Department of Hematology, Fifth Medical Center of Chinese PLA General Hospital, Beijing 100071, China, E-mail: ninghongmei72@sina.com

^＃^Wenshuai Zheng, Shenyu Wang and Yanchao Liang contributed equally to this article.

Journal: Annals of hematology

Supplementary Table 1 Multivariate analysis of overall survival in patients receiving RT or ST

| Era of diagnosis | Hazard ratio (95% CI) | | | |
| --- | --- | --- | --- | --- |
|  | RT | *P* | ST | *P* |
| Era1 | Reference |  | Reference |  |
| Era2 | 0.762 (0.614-0.946) | 0.014 | 0.831 (0.717-0.964) | 0.014 |

CI, confidence interval; RT, radiotherapy; ST, systemic therapy.

Supplementary Table 2 The demographics and clinical characteristics of patients ≥ 6-month survivors

| Clinical features | Overall  (n=10101) | RT  (n=2157) | ST  (n=2669) | CM  (n=541) | WW  (n=4734) |
| --- | --- | --- | --- | --- | --- |
| Era, n (%) |  |  |  |  |  |
| Era1 | 4371 (43.3) | 922 (42.7) | 1255 (47.0) | 337 (62.3) | 1857 (39.2) |
| Era2 | 5730 (56.7) | 1235 (57.3) | 1414 (53.0) | 204 (37.7) | 2877 (60.8) |
| Age, n (%) |  |  |  |  |  |
| 18-59 | 4120 (40.8) | 944 (43.8) | 1202 (45.0) | 287 (53.0) | 1687 (35.6) |
| 60-69 | 2763 (27.4) | 609 (28.2) | 731 (27.4) | 140 (25.9) | 1283 (27.1) |
| ≥ 70 | 3218 (31.9) | 604 (28.0) | 736 (27.6) | 114 (21.1) | 1764 (37.3) |
| Gender, n (%) |  |  |  |  |  |
| Female | 5097 (50.5) | 1061 (49.2) | 1369 (51.3) | 241 (44.5) | 2426 (51.2) |
| Male | 5004 (49.5) | 1096 (50.8) | 1300 (48.7) | 300 (55.5) | 2308 (48.8) |
| Stage, n (%) |  |  |  |  |  |
| Stage I | 6323 (62.6) | 1786 (82.8) | 1091 (40.9) | 353 (65.2) | 3093 (65.3) |
| Stage II | 3778 (37.4) | 371 (17.2) | 1578 (59.1) | 188 (34.8) | 1641 (34.7) |
| Grade, n (%) |  |  |  |  |  |
| Grade 1 | 4482 (44.4) | 936 (43.4) | 1163 (43.6) | 192 (35.5) | 2191 (46.3) |
| Grade 2 | 5619 (55.6) | 1221 (56.6) | 1506 (56.4) | 349 (64.5) | 2543 (53.7) |
| Extranodal disease, n (%) |  |  |  |  |  |
| No | 8022 (79.4) | 1612 (74.7) | 2248 (84.2) | 430 (79.5) | 3732 (78.8) |
| Yes | 2079 (20.6) | 545 (25.3) | 421 (15.8) | 111 (20.5) | 1002 (21.2) |
| Follow up time, n (%) |  |  |  |  |  |
| 6-59 months | 4036 (40.0) | 798 (37.0) | 1023 (38.3) | 136 (25.1) | 2079 (43.9) |
| ≥ 60 months | 6065 (60.0) | 1359 (63.0) | 1646 (61.7) | 405 (74.9) | 2655 (56.1) |
| Race/ethnicity, n (%) |  |  |  |  |  |
| NHB | 375 (3.7) | 52 (2.4) | 108 (4.0) | 16 (3.0) | 199 (4.2) |
| NHW | 8067 (79.9) | 1745 (80.9) | 2113 (79.2) | 437 (80.8) | 3772 (79.7) |
| NHA/PI | 526 (5.2) | 154 (7.1) | 115 (4.3) | 23 (4.3) | 234 (4.9) |
| Hispanic | 1133 (11.2) | 206 (9.6) | 333 (12.5) | 65 (12.0) | 529 (11.2) |

NHW, non-Hispanic white; NHB, non-Hispanic black; NHA/PI, non-Hispanic Asian or Pacific Islander; RT, radiotherapy; ST, systemic therapy; CM, combined modality; WW, watch and wait.

Supplementary Table 3 Multivariate analysis of second primary malignancies risk in patients receiving RT or ST

| Era of diagnosis | Relative risk (95% CI) | | | |
| --- | --- | --- | --- | --- |
|  | RT | *P* | ST | *P* |
| Era1 | Reference |  | Reference |  |
| Era2 | 0.174 (0.129-0.233) | < 0.001 | 0.365 (0.283-0.468) | < 0.001 |

CI, confidence interval; RT, radiotherapy; ST, systemic therapy.
